# Hsa‐miR‐409‐3p regulates endothelial progenitor senescence via PP2A‐P38 and is a potential ageing marker in humans

**DOI:** 10.1111/jcmm.17691

**Published:** 2023-02-09

**Authors:** Yi‐Nan Lee, Yih‐Jer Wu, Hsin‐I Lee, Hsueh‐Hsiao Wang, Chung‐Lieh Hung, Chiung‐Yin Chang, Yen‐Hung Chou, Ting‐Yi Tien, Chun‐Wei Lee, Chao‐Feng Lin, Cheng‐Huang Su, Hung‐I Yeh

**Affiliations:** ^1^ Department of Medical Research MacKay Memorial Hospital Taipei City Taiwan; ^2^ Division of Cardiology/Cardiovascular Center MacKay Memorial Hospital Taipei City Taiwan; ^3^ Mackay Medical College New Taipei City Taiwan; ^4^ MacKay Junior College of Medicine, Nursing and Management Taipei Taiwan

**Keywords:** angiogenesis, biomarker, endothelial progenitor cells, hsa‐miR‐409‐3, protein phosphatase 2A, senescence

## Abstract

We explored the roles of hsa‐microRNA (miR)‐409‐3p in senescence and signalling mechanism of human endothelial progenitor cells (EPCs). Hsa‐miR‐409‐3p was found upregulated in senescent EPCs. Overexpression of miRNA mimics in young EPCs inhibited angiogenesis. In senescent EPCs, compared to young EPCs, protein phosphatase 2A (PP2A) was downregulated, with activation of p38/JNK by phosphorylation. Young EPCs treated with siPP2A caused inhibited angiogenesis with activation of p38/JNK, similar to findings in senescent EPCs. Time series analysis showed, in young EPCs treated with hsa‐miR‐409‐3p mimics, PP2A was steadily downregulated for 72 h, while p38/JNK was activated with a peak at 48 hours. The inhibited angiogenesis of young EPCs after miRNA‐409‐3p mimics treatment was reversed by the p38 inhibitor. The effect of hsa‐miR‐409‐3p on PP2A signalling was attenuated by exogenous VEGF. Analysis of human peripheral blood mononuclear cells (PBMCs) obtained from healthy people revealed hsa‐miR‐409‐3p expression was higher in those older than 65 years, compared to those younger than 30 years, regardless of gender. In summary, hsa‐miR‐409‐3p was upregulated in senescent EPCs and acted as a negative modulator of angiogenesis via targeting protein phosphatase 2 catalytic subunit alpha (PPP2CA) gene and regulating PP2A/p38 signalling. Data from human PBMCs suggested hsa‐miR‐409‐3p a potential biomarker for human ageing.

## 1 INTRODUCTION

Endothelial progenitor cells (EPCs), obtained from peripheral blood and identified as CD34 antigen‐positive (CD34^+^) mononuclear cells, were marrow‐derived stem cells and can differentiate into endothelial cells to promote neovascularisation in response to ischemic injury.^1^ Cell therapy using EPCs has been shown beneficial in ischaemia‐related cardiovascular diseases (CVD) and emerged as useful substrates for neovascularization.^2^, ^3^, ^4^ However, some limitations make their clinical application difficult, such as heterogeneity in progenitor cell types, lack of standardization of specific surface markers and reduced number during ageing.^5^, ^6^, ^7^ Nonetheless, the angiogenic potential of EPCs has been an important target in regenerative medicine.^8^, ^9^, ^10^

Numerous studies indicated that microRNA (miR or miRNA) is involved in post‐transcriptional regulation of gene expression concerning diverse biological functions, including ageing and angiogenesis.^11^, ^12^, ^13^ A previous report showed that angiogenesis and tissue repair were regulated by miRNA‐135a‐3p via targeting p38 signalling in endothelial cells, revealing a link among miRNA, angiogenesis and endothelial cells.^14^ In addition, increased miRNA‐183‐5p with age was involved in stem cell senescence.^15^ Furthermore, several studies have also addressed the regulation of miRNA during culture‐induced senescence of vascular cells or in tissues.^16^, ^17^, ^18^ These findings suggested that senescence and miRNAs may play an integrated role in modulating the pathologic processes of human CVD via the regulation of progenitor cell activity. We, therefore, in the present study explored the impact of miRNA in human EPC senescence and its signalling mechanism. An established serial passage model was applied to reflect the senescence process of EPCs during in vitro expansion.^7^ In addition, we collected blood from young and old people to examine the potential of clinical application.

## 2 MATERIALS AND METHODS

### 2.1 Study population and ethics statement

Fifty‐seven healthy volunteers were recruited from 2018 to 2019 at the Mackay Memorial Hospital, Taipei, Taiwan. All participants underwent a medical history review, routine physical examination and blood test (Table 1). Hypertension was diagnosed if patients were with systolic blood pressure (BP) ≥ 140 mmHg and/or diastolic BP ≥90 mmHg or took medication for hypertension. Diabetes was diagnosed if patients were with haemoglobin A1C ≧ 6.5% or with classic symptoms of hyperglycaemia, random plasma glucose ≧200 mg/dL. Additionally, hyperlipidemia was diagnosed if patients were with fasting triglyceride levels ≥150 and ≤500 mg/dL, and LDL cholesterol >130 mg/dL. The study was conducted according to the principles outlined in the Declaration of Helsinki and approved by the ethics committee of the MacKay Memorial Hospital (#19MMHIS223e). Informed consent was given before the collection of venous blood (80 mL).

**TABLE 1:** Clinical characteristics of study participants

	<30 years (*n* = 18)	>65 years (*n* = 39)	*p* value
Male/Female, *n*	13 /5	19 /20	
Age (years), mean ± SEM	25.8 ± 0.9	69.7 ± 0.5	<0.001
Body mass index (kg/m^2^), mean ± SEM	24.4 ± 2.4	24.4 ± 3.5	0.996
Biochemical data, mean ± SEM			
Fasting plasma glucose (mg/dL)	88.6 ± 2.3	106.5 ± 3.2	0.009
Total cholesterol (mg/dL)	181.4 ± 8.4	197.8 ± 7.2	0.240
HDL cholesterol (mg/dL)	52.3 ± 2.5	57.0 ± 2.4	0.272
LDL cholesterol (mg/dL)	123.2 ± 15.1	106.9 ± 6.6	0.308
Triglycerides (mg/dL)	103.8 ± 15.7	107.9 ± 7.1	0.792
GPT (U/L)	22.8 ± 5.1	31.3 ± 5.6	0.482
GOT (U/L)	21.1 ± 1.3	26.9 ± 1.5	0.047
BUN (mg/dL)	12.2 ± 0.8	15.8 ± 1.2	0.156
Creatinine (mg/dL)	0.83 ± 0.06	0.82 ± 0.03	0.830
Health history, *n* (%)			
Alcohol drinker	5 (28.8%)	6 (15.4%)	0.263
Current smoker	0 (0%)	0 (0%)	1.000
Hypertension	0 (0%)	18 (46.2%)	<0.001
Diabetes mellitus	0 (0%)	7 (17.9%)	0.174
Hyperlipidemia	0 (0%)	8 (20.5%)	0.089
Drug therapy, *n* (%)			
Anti‐hyperlipidemia	0 (0%)	0 (0%)	1.000
Anti‐diabetics	0 (0%)	7 (17.9%)	0.174
Anti‐hypertension	0 (0%)	18 (46.2%)	<0.001

*Note*: Data are mean ± SEM and analysed using unpaired Student's *t* test for comparison between groups.

*Abbreviations*: BUN, blood urea nitrogen; GOT, aspartate aminotransferase; GPT, alanine aminotransferase; HDL, high‐density lipoprotein; LDL, low‐density lipoprotein.

### 2.2 Human CD34‐positive EPC isolation

Peripheral blood mononuclear cells (PBMCs) were isolated from the buffy coat and purified by Ficoll‐Paque™ PLUS (GE Healthcare) according to the manufacturer's instruction. The cells were then washed with 0.9% normal saline and centrifuged at 200–400 × *g* for 5 min.^19^ CD34‐positive EPCs were isolated from the PBMCs using a CD34 MicroBead kit and MACS Cell Separation System (all from Miltenyi Biotec). Finally, EPCs were resuspended in Endothelial Cell Growth Medium MV2 (PromoCell) with supplementMix (PromoCell) and cultured on the six‐well plate (Corning) coated with 1 μg/cm^2^ of human fibronectin (Corning) in a 5% CO_2_ incubator at 37°C.^19^ EPC colonies displayed cobblestone‐like morphology after culturing in MV2 medium for 10–14 days then were trypsinized with 0.05% trypsin–EDTA (Gibco) for passage.^19^ Young EPCs were maintained within 10 passages for experiments. Senescent EPCs were obtained after serial propagation and defined as the doubling time of more than 2 folds of the young EPCs.

### 2.3 RNA isolation and quantitative real‐time‐PCR (qRT‐PCR) analysis

Total RNA from EPCs was extracted using Quick‐RNA Miniprep Plus kit (Zymo Research) according to the manufacturer's instructions. For mRNA analysis, 1 μg of RNA was applied to the reaction of reverse transcription using iScript cDNA Synthesis kit (BioRad). One miceolitre of the cDNA products was used for quantitative PCR amplification. For microRNA analysis, first‐strand cDNA was synthesized at 37°C for 1 h followed by 85°C incubation for 5 min using the Mir‐X™ miRNA First‐Strand Synthesis kit (Clontech Laboratories, Inc.). All PCR reactions were performed using ABI StepOne Plus Real‐Time PCR System (Applied Biosystems; Thermo Fisher Scientific, Inc.). The levels of miR‐409‐3p were normalized with RNU6. The miR‐409‐3p forward primer (cas. no. HmiRQP0484) was purchased from GeneCopoeia, Inc and the other primers were provided by the Mir‐X miRNA qRT‐PCR kit (Clontech Laboratories, Inc.). The levels of PPP2CA mRNA were normalized by the level of β‐actin. The specific primers were used as follows: PPP2CA forward: 5′‐CTGCAATCATGGAACTTGACG‐3′ and reverse, 5′‐ACTGTTGCTCTTCCCATTTCC‐3′; β‐actin forword: 5′‐CCTCCCTGGAGAAGAGCTACGA‐3′ and reverse, 5′‐CGCCAGACAGCACTGTGTTG‐3′.

### 2.4 Next‐generation sequencing (NGS) and analysis

**FIGURE 1:**
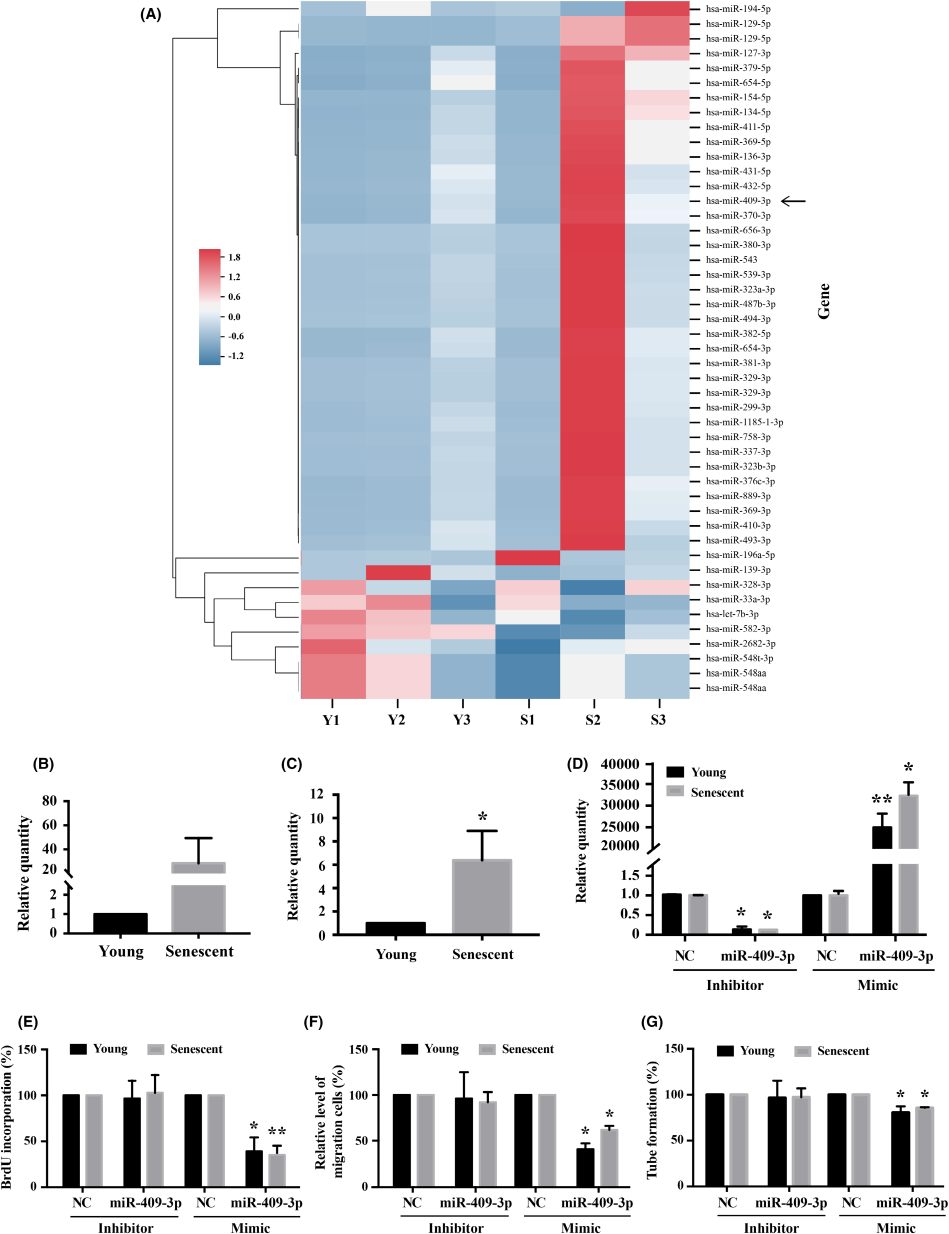
Analysis of senescence‐associated microRNA in human EPCs and measurement of cellular activities 48 h after anti‐miRNA and miRNA mimics treatment. (A) Screening of senescence‐associated microRNA in human EPCs using Next Generation Sequencing (NGS) analysis. Heat map of miRNA expression data obtained from three human donors. Relative miRNA expression was depicted according to the colour scale shown. Red indicated upregulation and grey indicated downregulation. The young (Y) EPCs (Y1–Y3) represented three individual repeats, along with the corresponding senescent (S) EPCs (S1–S3). Arrow, hsa‐miR‐409‐3p. (B) Expression of hsa‐miR‐409‐3p was enhanced (>40 folds) in senescent EPCs compared to young cells (*n* = 3). (C) qRT‐PCR confirmed the enhanced expression of hsa‐miR‐409‐3p in the senescent EPCs, compared to young EPCs (*n* = 8). (D) Reduced and enhanced expression of hsa‐miR‐409‐3p was detected using qRT‐PCR in young and senescent EPCs transfected with each of miR‐409‐3p inhibitor and mimics (*n* = 4). (E–G) Transfection with miR‐409‐3p inhibitor minimally affected the cellular activities of both young and senescent cells, including (E) proliferation detected using BrdU assay, (F) migration using Boyden Chamber assay, and (G) tube formation activity using matrigel. In contrast, transfection with miR‐409‐3p mimics attenuated all 3 activities. *n* = 5 and *n* = 9 respectively for each bar in miRNA inhibitor groups and miRNA mimics groups. Data are mean ± SEM and analysed using unpaired Student's *t* test for comparison between groups (C), and one‐way anova followed by post hoc Fisher's least significant difference test for multiple group comparisons (D–G). *, *p* < 0.05, **, *p* < 0.01 vs. corresponding control. Ctrl, control. NC, negative control

Endothelial progenitor cells (EPCs) were harvested for total RNA extraction using Trizol® Reagent (Invitrogen). Extracted RNAs were measured the OD260/OD280 absorbance ratio to evaluate RNA quality by ND‐1000 spectrophotometer (Nanodrop Technology). Small RNA sequencing was performed by Welgene Biotechnology Company at the Solexa platform with a single‐end sequencing method in 75 nucleotides read length. Differentially expressed miRNAs greater than 2.0‐fold changes between young and senescent cell groups were indicated with reads per million (RPM) > 10 (Figure 1A).^20^

### 2.5 miRNA and short interference RNA (siRNA) transfection

All miRNAs and siRNAs were transfected by LipofectAMINE 3000 (Invitrogen). EPCs were grown on 1% gelatin‐coated petri dishes and kept in the range of 70 to 80% confluency for routine experiments. Commercial products of miR‐409‐3p mimic (Ambion # 4463066) and inhibitor along with their negative controls (Ambion # 4464058) were purchased from ThermoFisher Scientific Inc. Chemically synthesized PPP2CA‐specific siRNA was purchased from Integrated DNA Technologies. Nonsense siRNA (cat. no. 5‐01‐14‐03; in Figure 3A–I), mirVANA miRNA inhibitor negative control (Ambion # 4464084, Figure 1D–G) and mimic negative control (Figure 1D–G, Figure 4A–G, and Figure 5A–C) were used as a transfection control in the individual experiments. After 5.5 h of incubation at the time of transfection, miRNA mixtures were replaced with 20% FBS MV2 for recovery. After 24 h recovery, EPCs were treated or untreated VEGF 50 ng/mL (R&D Systems) overnight.^21^, ^22^

### 2.6 Cell proliferation, migration and tube formation assay

Angiogenic activities were performed at 48 h post‐LipofectAMINE 3000‐mediated transfection.

Cell proliferation was determined by using a bromodeoxyuridine (BrdU) cell proliferation assay kit (Calbiochem) to detect BrdU incorporation into DNA duplex during cell proliferation. Young and senescent EPCs were seeded 10^4^ cells in 500 μL media overnight. The levels of incorporated BrdU were measured by a SpectraMAX 190 absorbance microplate reader (Molecular Devices) at dual wavelengths from 450 to 540 nm.^23^

Cell migration was evaluated by Boyden chamber assay in 24‐well transwell chambers (8 μm pore size, Corning). The transwell assay was based on a chamber of two medium‐filled compartments separated by a microporous membrane. EPCs (3 × 10^4^ cells in 100 μL of 0.5% fetal bovine serum (FBS) medium MV2) were seeded in the upper compartment, whereas the lower compartment was loaded with 600 μL of supernatants from EPCs post‐48 h of transfection. After incubation at 37°C for 4 h, the membrane between the two compartments was fixed (−20°C methanol for 5 min) and stained with 18.7 mM bisbenzimide (Sigma; for 20 min). After PBS washes, the number of EPCs that had migrated into the lower side of the membrane was determined by applying inverted fluorescence microscopy (Leica) at ×50 magnification and analysed by Leica QWin image analysis software (Version number: V3.5.2) to determine the average number of cells transmigration.^23^

For tube formation assay, 200 μL Matrigel (BD Biosciences) was added onto the 24‐well plate for 30 min to allow the gel to solidify. Cells were resuspended in MV2 containing 2.5% FBS, seeded at a density of 3 × 10^4^ cells per well and incubated at 37°C for 24 h to form capillary‐like structures. The cumulative tube length was calculated in four randomly selected microscopic fields at 50× magnification derived from four independent experiments using Leica QWin image analysis software (Cambridge, UK, Version number: V3.5.2) as an indicator of their angiogenic potential.^23^

### 2.7 Luciferase reporter assay

The target sequence of miR‐409‐3p in PPP2CA 3′‐UTR or its mismatched version was cloned into the pmirGLO vector (Promega). HEK293 cells were transiently transfected with vectors bearing miR‐409‐3p mimic or its mismatched version. Cell lysates were harvested for measuring firefly and renilla luciferase activity using the Dual‐Luciferase Reporter Assay System according to the manufacturer's manual (Promega). Luciferase activity (firefly/Renilla luciferase activity) of each vector was normalized with the group of negative control.

### 2.8 Western blot analysis

Endothelial progenitor cells (EPCs) were washed and lysed with RIPA buffer (Sigma‐Aldrich). Protein concentrations were determined by using Pierce BCA Protein Assay Kit (Thermo Fisher Scientific, Inc). Aliquots of cell lysates were loaded into 10% SDS‐polyacrylamide gels, electrophoresed and transblotted onto PVDF membranes (Bio‐rad). The blots were blocked with 10× blocking buffer (Sigma‐Aldrich) for 1 h and incubated with primary antibodies (1–1000 dilution) at 4°C overnight.^24^ The antibodies included phosphorylated p38 mitogen‐activated protein kinase (p‐p38), total p38, phosphorylated c‐Jun N‐terminal kinase (p‐JNK), total JNK, phosphorylated extracellular signal‐regulated kinase (p‐ERK) and total ERK from Cell Signalling Technology. The other antibodies included PP2A from Arigo Biolaboratories. The other antibodies of loading control included GAPDH and α‐tubulin from Thermo Fisher Scientific Inc. and β‐actin from Sigma‐Aldrich. The blots were further incubated with horseradish peroxidase‐conjugated secondary antibodies (1–10,000 dilution, Jackson ImmunoResearch) for 1 h at room temperature. Immunoreactivity was visualized using VisioGlo (Amresco) according to the manufacturer's instructions. The radiographs were subject to VisionWorks software (Ultra‐Violet Products Ltd.). To normalize the expression level, blots were stripped with stripping buffer (69 mmol/L SDS, 100 mmol/L 2‐mercaptoethanol, 93.75 mmol/L Tris‐HCl, pH 6.8) at 56°C and incubated with anti‐β‐actin, GAPDH or α‐tubulin antibody as internal control.^24^

### 2.9 Immunofluorescence

After miR‐409‐3p mimics and siRNAs transfection for 48 h, EPCs were seed on 2% gelatin‐coated cover slides and matrix gel (354,230; Corning) overnight and then fixed with 4% paraformaldehyde (electron‐microscopy grade, Electron Microscopy Sciences) for 10 min at room temperature. Cells were washed with 0.2% Triton X‐100‐PBS for three times and blocked with 10% horse serum for 1 h. The following antibodies were used: total p38 (9212; Cell Signalling Technology) and p‐p38 (9212; Cell Signalling Technology). Antibodies were diluted at 1:100 and incubated at 4°C overnight. For cells on slides, corresponding secondary antibodies were incubated at room temperature for 3 h. For cells on matrix gel, corresponding secondary antibodies were incubated in 0.5% Triton X‐100‐PBS at 4°C overnight. After three times of washes, the walls of chamber slides were removed and mounted with ProLong™ Glass Antifade Mountant (Thermo, P36984). Images were acquired by Leica SP8 Laser Confocal Microscope (Germany).

### 2.10 Statistical analysis

All experiments' data are expressed as mean ± SEM. Experimental groups were compared for statistical significance using an unpaired Student's *t* test for comparison between two groups or using a one‐way analysis of variance (anova) test, followed by a Fisher's least significant difference test for multiple comparisons if the number of study groups was three or more. A *p* value less than 0.05 indicates statistical significance. The n number quoted refers to the number of separate experiments. All experiments were performed at least three times.

## 3 RESULTS

### 3.1 Identification of senescence‐associated microRNA and validation of its role in human EPCs

Human senescent EPCs were defined as having an increase of cell doubling time more than two‐folds of the young cells.^7^ SA‐β‐Gal assay showed that absorbance was increased by more than 100% in the senescent cells.^7^ Additionally, the telomere length was significantly decreased in the senescent cells.^7^ Screen of microRNAs using NGS analysis showed that the expression of hsa‐miR‐409‐3p was enhanced in the senescent EPCs, compared to the young EPCs (Figure 1A,B). The significantly enhanced expression of hsa‐miR‐409‐3p in the senescent EPCs from eight different human donors was further confirmed using qRT‐PCR (Figure 1C). Transfection using lipofectamine 3000 with miR‐409‐3p inhibitor significantly downregulated the level and that with miRNA‐409‐3p mimics upregulated the level (Figure 1D).

Regarding the cellular effects of different microRNAs levels in young and senescent EPCs, overexpression of hsa‐miR‐409‐3p significantly attenuated proliferation (Figure 1E), migration (Figure 1F) and tube formation (Figure 1G) activities, while no change of the activities was observed after transfection of miR‐409‐3p inhibitor (Figure 1E–G).

### 3.2 Prediction of binding site of hsa‐miR‐409‐3p for protein phosphatase 2 catalytic subunit alpha (PPP2CA) gene and analysis of protein phosphatase 2A (PP2A) expression

**FIGURE 2:**
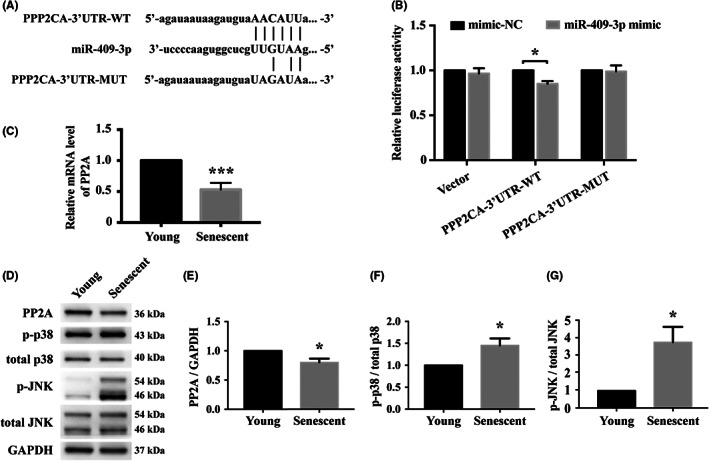
Prediction of binding site of hsa‐miR‐409‐3p for protein phosphatase 2 catalytic subunit alpha (PPP2CA) gene and examination of its product protein phosphatase 2A (PP2A). (A) Diagram showing the seed sequences of hsa‐miR‐409‐3p and its predicted binding site in the 3’‐UTR of PPP2CA gene by prediction methods including TargetScan and miRanda. (B) Luciferase reporter assays of human embryonic kidney cells 293 co‐transfected with vector, wild‐type 3’‐UTR of PPP2CA (PPP2CA‐3’UTR‐WT) or mutant 3’‐UTR of PPP2CA (PPP2CA‐3’UTR‐MUT), along with mimic negative control (NC) or miR‐409‐3p mimics showed reduced activity only in the group co‐transfected with PPP2CA‐3’UTR‐WT and miR‐409‐3p mimics. Concordantly, in senescent cells, PP2A expression was downregulated at both (C) transcript and (D and E) protein levels, with activation of (F) phosphorylated p38 (p‐p38) and (G) phosphorylated JNK (p‐JNK). *n* = 4 for each bar. Data are mean ± SEM and analysed using unpaired Student's *t* test for comparison between groups. *, *p* < 0.05, ***, *p* < 0.001 vs. corresponding control. p38, p38 mitogen‐activated protein kinase. JNK, c‐Jun N‐terminal kinase

To explore miRNA‐binding sites to gain an insight into the target gene regulation, both the TargetScan and miRanda web servers were checked. The results demonstrated the seed sequences of hsa‐miR‐409‐3p and its predicted binding site was located in the 3’‐UTR (untranslated region) of the PPP2CA gene (Figure 2A). Luciferase reporter assays in human embryonic kidney cells 293 (HEK293 cells) using lipofectamine‐mediated transfection showed that co‐transfection with miR‐409‐3p mimics and wild‐type 3’‐UTR of PPP2CA, but not mutant 3’‐UTR of PPP2CA, led to decreased luciferase activity, compared to mimic negative control (Figure 2B). In senescent EPCs during serial passage, PP2A was downregulated at the transcript level (Figure 2C) and protein level (Figure 2D,E). In addition, both phosphorylated p38 (p38 mitogen‐activated protein kinase) and phosphorylated JNK (c‐Jun N‐terminal kinase) were upregulated (Figure 2F,G).

### 3.3 Reduction of angiogenic activity of human EPCs treated with PP2A siRNA (siPP2A) and the signalling pathway

**FIGURE 3:**
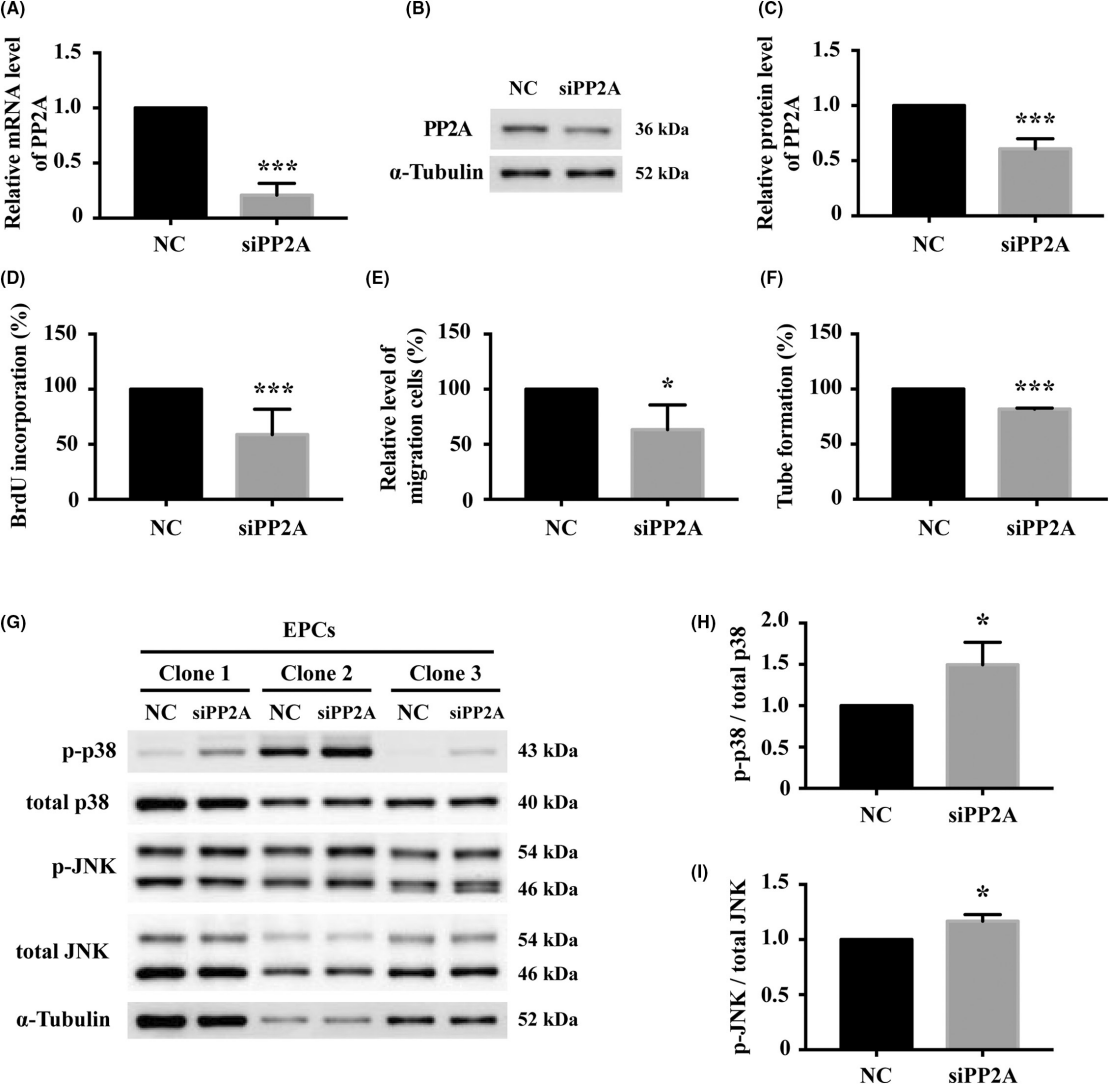
Angiogenic activities of young human EPCs after treatment with siRNA specific to PP2A (siPP2A) and western blot analysis of young human EPCs treated with PP2A siRNA. Note that (A) transcript and (B and C) protein expression levels of PP2A were suppressed in siPP2A‐treated EPCs, accompanied by attenuated cellular activities of (D) proliferation, (E) migration and (F) tube formation. *n* = 3 for each bar. (G) In three different clones of EPCs, phosphorylated p38 (p‐p38) and phosphorylated JNK (p‐JNK) were upregulated after siPP2A treatment. Quantitative Analysis confirmed the activation of (H) p38 and (I) JNK signalling pathways. *n* = 3 for each bar. Data are mean ± SEM and analysed using unpaired Student's *t* test for comparison between groups. *p < 0.05, ***p < 0.001 vs. corresponding negative control (NC). Abbreviations are as in Figure 2

To investigate the relation between PP2A expression and cellular function, young EPCs were treated with siPP2A, which suppressed PP2A significantly at both transcript and protein levels (Figure 3A–C) and attenuated proliferation (Figure 3D), migration (Figure 3E) and tube formation (Figure 3F) of the cells. Western blot analysis of young EPCs treated with PP2A siRNA (Figure 3G) demonstrated activation of p38 and JNK (Figure 3H,I). The above findings suggested a strong relation among hsa‐miR‐409‐3p, PP2A and senescence‐associated angiogenic dysfunction in human EPCs and involved activation of p38 and JNK.

### 3.4 Validation of the relation between miRNA‐409‐3p expression and PP2A regulation in human EPCs with or without exogenous vascular endothelial growth factor (VEGF)

**FIGURE 4:**
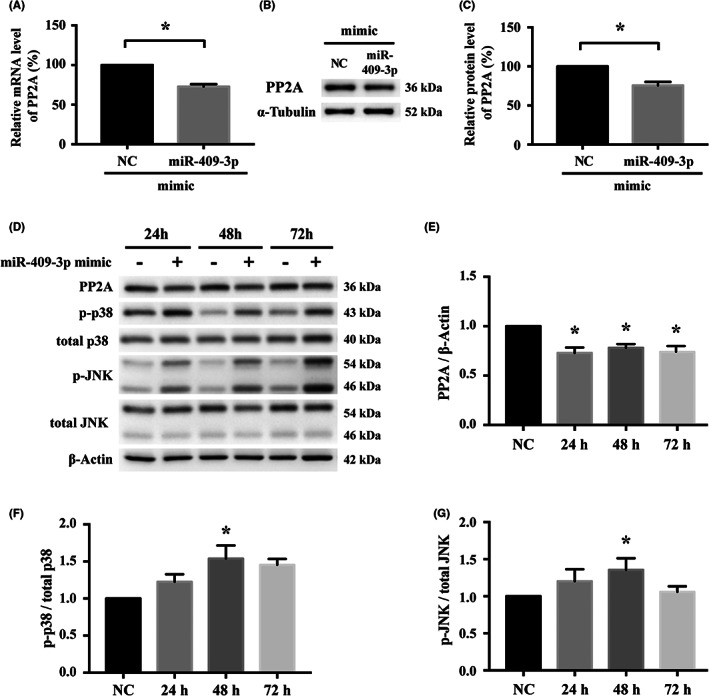
Regulation of PP2A by miR‐409‐3p and the signalling proteins in young human EPCs. PP2A was downregulated by miR‐409‐3p mimic at (A) transcript and (B and C) protein levels, compared to corresponding NC. (D) Time series analysis showed, in cells treated with the mimic, (E) PP2A was downregulated for at least 72 h, while (F) p‐p38 and (G) p‐JNK were activated with peaks at 48 h. *n* = 5 for each bar. Data are mean ± SEM and analysed using one‐way anova followed by post hoc Fisher's least significant difference test for multiple group comparisons. *p < 0.05 vs. NC. Abbreviations are as in Figure 2

PP2A was significantly downregulated by miR‐409‐3p mimics at both transcript (Figure 4A) and protein levels (Figure 4B,C) in young EPCs, compared to the negative control. Time series analysis demonstrated that PP2A protein expression was significantly downregulated for at least 72 h after the mimics treatment (Figure 4D,E). Concomitantly, activations of p38 and JNK was noted and reached a peak at 48 h after the mimics treatment (Figure 4D,F,G). Therefore, regulation of PP2A in human EPCs by hsa‐miR‐409‐3p and involvement of p38 and/or JNK signalling pathway were further confirmed.

Concerning the effect of VEGF on the regulatory system of miRNA‐409‐3p signalling in EPCs, the results showed that, with overexpression of hsa‐miR‐409‐3p followed by adding VEGF, the regulatory trends by PP2A (Figure S1A,B), p‐p38 (Figure S1A,C) and p‐JNK (Figure S1A,D) were not changed, though the difference in PP2A became insignificant. In siPP2A‐treated EPCs followed by adding VEGF, the regulatory trends by PP2A (Figure S1E,F) and p‐p38 (Figure S1E,G) also remained unchanged, but the difference became insignificant. In contrast, the change of p‐JNK by siPP2A after adding VEGF nearly disappeared (Figure S1E,H). Additionally, our results showed that tube formation of EPCs was inhibited by overexpression of hsa‐miR‐409‐3p. The inhibited trend remained unchanged after adding VEGF (Figure S2A,B). In siPP2A‐treated EPCs, tube formation was inhibited. The decreased trend of tube formation remained unchanged after adding VEGF but became insignificant (Figure S2C,D). As a result, VEGF was involved in the PP2A signalling. The angiogenesis‐associated signalling molecule, ERK, was also tested to investigate its relationship to hsa‐miR‐409‐3p. The result showed that the protein expression level of p‐ERK was minimally changed by overexpression of hsa‐miR‐409‐3p, which implied ERK was not involved in the regulation of hsa‐miR‐409‐3p (Figure S3A,B).

### 3.5 Verification of angiogenic alternation and plausibility of signalling pathway in miRNA‐409‐3p‐overexpressed EPCs

**FIGURE 5:**
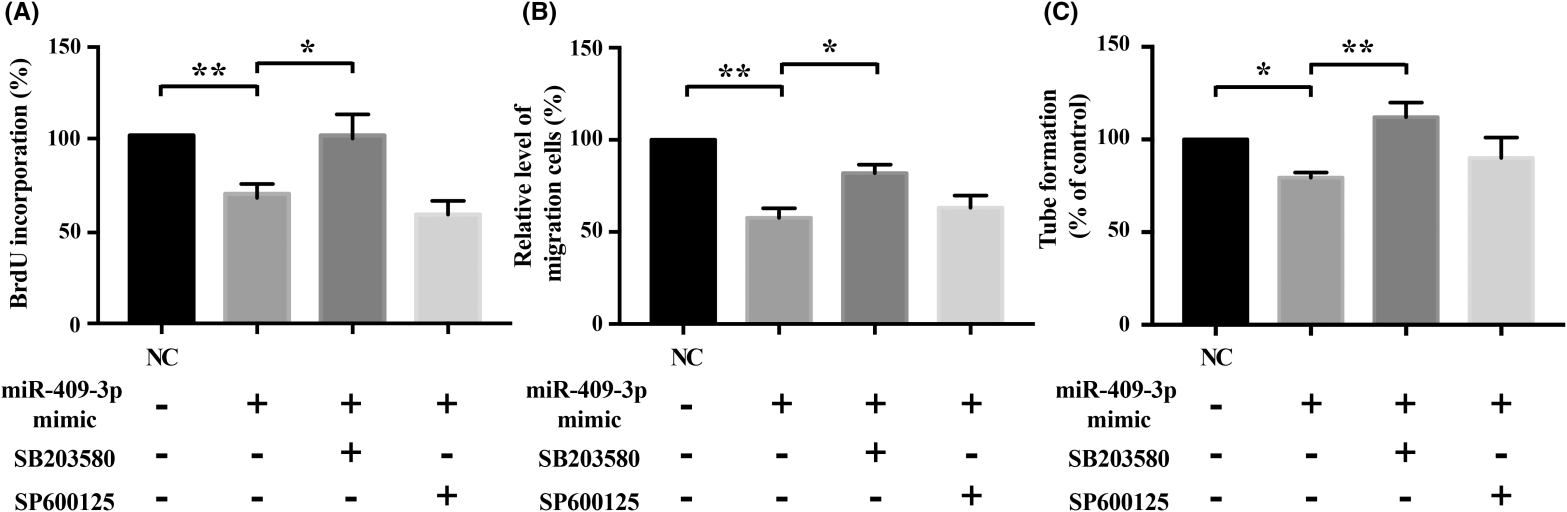
Angiogenic regulation of miRNA‐409‐3p‐overexpressed young EPCs treated with signalling pathway inhibitors. Inhibited (A) proliferation, (B) migration and (C) tube formation activities in miRNA‐409‐3p‐overexpressed EPCs were significantly reversed by adding SB203580 (P38 inhibitor), but not by SP600125 (JNK inhibitor), compared to negative control (NC). Data are shown as mean ± SEM. Statistical significance was determined using one‐way anova followed by post hoc Fisher's least significant difference test for multiple group comparisons. *n* = 5. *p < 0.05, **p < 0.01 vs. corresponding control. Abbreviations are as in Figure 2

To make sure the signalling pathway is activated by miRNA‐409‐3p overexpression, the young EPCs were treated with signalling pathway inhibitors. The results showed that inhibited proliferation (Figure 5A), migration (Figure 5B) and tube formation (Figure 5C) activities in miRNA‐409‐3p‐overexpressed EPCs were all reversed by p38 inhibitor SB203580 (Figure 5A–C), but not by JNK inhibitor SP600125, though partial recovery of tube formation activity was seen by SP600125. Therefore, p38 pathway was involved in the regulation of senescence‐related EPC dysfunction.

Moreover, after overexpression of hsa‐miR‐409‐3p in EPCs, immunofluorescence staining demonstrated co‐localization of the key phospho‐signal transmitter (phosphorylated p38 (p‐p38)) in nuclei, by using cell culture (panel h, Figure S4A) and matrigel (panel j, Figure S4B).

### 3.6 Age‐dependent expression levels of hsa‐miR‐409‐3p in human PBMCs

**FIGURE 6:**
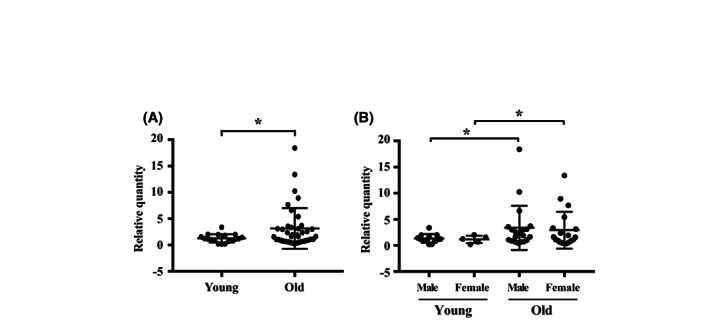
Dot plots analysis of hsa‐miR‐409‐3p transcript level in human peripheral blood mononuclear cells using qRT‐PCR. The level was significantly higher (A) in the old group (> 65 years, *n* = 39) compared to the young group (<30 years, *n* = 18), and (B) in both males (*n* = 20) and females (*n* = 19) >65 years old (old group) compared to the corresponding young group (< 30 years, *n* = 13 in male and *n* = 5 in female) of the same gender. Data are shown as mean ± SD. Statistical significance was determined using Student's *t* test for comparison between two groups, and one‐way anova followed by post hoc Fisher's least significant difference test for multiple group comparisons. *p < 0.05 vs. corresponding control

We collected venous blood from 57 healthy people and determined the hsa‐miR‐409‐3p expression level of PBMCs using qRT‐PCR. The clinical characteristics of young (<30 years) and old (>65 years) groups are shown in Table 1, which revealed age, fasting plasma glucose, aspartate aminotransferase (GOT) and percentage of patients with hypertension and those taking anti‐hypertensive drugs were different between the groups. The average hsa‐miR‐409‐3p expression level in PBMCs of the old group was significantly higher than that of the young group (Figure 6A) and the difference was regardless of gender (Figure 6B).

**FIGURE 7:**
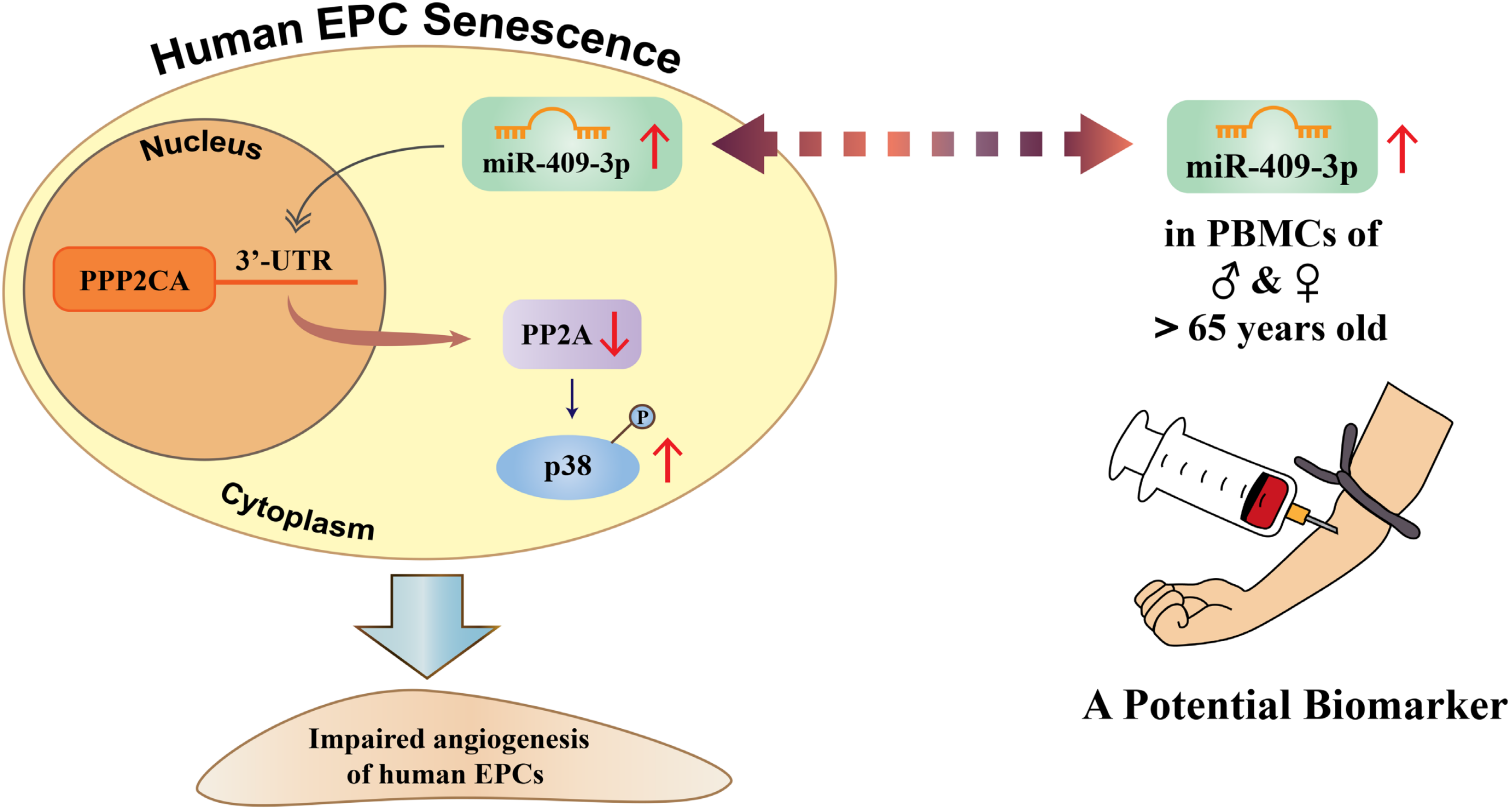
Regulation of hsa‐miR‐409‐3p in human senescent EPCs and its clinical implication. Hsa‐miR‐409‐3p with its binding site in the 3’‐UTR of PPP2CA gene was upregulated in senescent EPCs and acted as a negative modulator of angiogenesis via targeting PPP2CA gene and regulating PP2A/p38 signalling. Using a cut‐off of 65 years for old participants, hsa‐miR‐409‐3p expression in PBMCs is significantly upregulated in participants older than 65 years, compared to the young group under 30 years, regardless of gender. hsa‐miR‐409‐3p is a potential biomarker for human ageing. Abbreviations are as in Figure 2

The regulation by hsa‐miR‐409‐3p, the related signalling pathways, epigenetic modification of angiogenic activities in human EPCs, and clinical implications were summarized in Figure 7.

## 4 DISCUSSION

Three interconnected questions were raised and answered in the present study: (1) What is the senescence‐associated miRNA in human EPCs? (2) What is the signal transduction underlying the miRNA‐directing EPC senescence? (3) What is the clinical implications of the senescence‐associated miRNA of human EPCs? By NGS analysis, we demonstrated for the first time that hsa‐miR‐409‐3p is an important senescence‐associated miRNA in human EPCs. The signal transduction involved PP2A and p38 to regulate angiogenesis of EPCs. Moreover, hsa‐miR‐409‐3p in PBMCs can be a potential human biomarker of human ageing, as the expression level was significantly higher in the individuals >65 years old, compared to those <30 years.

To compare the biological difference between young and senescent EPCs in terms of expression of miRNA, in this report, human senescent EPCs were defined as the EPCs with replication‐induced senescence at a modest level. Compared with young EPCs, senescent EPCs were defined to have an increase in the cell doubling time more than two‐folds of the young cells, accompanied by significant shortening of telomere length and an increase of up to 130% in acidic *β*‐galactosidase activity.^7^ Senescence process during replication jeopardizes angiogenic function of EPCs by which cell therapy requires an adequate number of cells with proper cellular activities. Since miRNAs have recently emerged as important regulators of cellular senescence and ageing,^13^, ^25^ in the present study, NGS analysis was utilized to screen and determine the sequence variation of miRNA between young and senescent human EPCs.^26^, ^27^ After confirmation by qRT‐PCR, hsa‐miR‐409‐3p was noted to be upregulated in senescent human EPCs compared to young EPCs. In the literature, miR‐409‐3p was reported to be expressed by embryonic stem cells and to regulate tumour cell invasion/proliferation^28^, ^29^ and haemostasis.^30^, ^31^ In our study, it is the first time that hsa‐miR‐409‐3p was linked to dysfunctional angiogenesis, including proliferation, migration and tube formation, in human EPCs. Moreover, combined results from microRNA target prediction tools showed that the seed sequences of hsa‐miR‐409‐3p with its predicted binding site were located in the 3’‐UTR of PPP2CA gene which regulates the expression of PP2A protein, as confirmed by the luciferase reporter assay. PP2A protein encoded by the PPP2CA gene is a major kind of serine/threonine protein phosphatase in eukaryotic cells and plays important roles in the regulation of cell cycle and signal transduction.^32^, ^33^ In our study, PP2A protein expression was downregulated along with upregulated phospho‐p38 and phospho‐JNK in senescent EPCs during serial passage. Furthermore, in young human EPCs treated with siPP2A, the angiogenic activity was significantly decreased in proliferation, migration and tube formation. These findings were also compatible with the declined angiogenesis in senescent EPCs with more expression of miR‐409‐3p. Together with hsa‐miR‐409‐3p and its binding site in the 3’‐UTR of PPP2CA gene, the results indicated a close link between hsa‐miR‐409‐3p and PP2A, causing senescence‐associated angiogenic dysfunction in human EPCs. In the literature, there has been no report showing the relationship between miR‐409‐3p and PP2A, particularly with a biological effect on human EPCs.

Concerning the signalling mechanism dominating the senescence‐related dysfunctional angiogenesis in EPCs, western blot analysis of signalling protein treated with siPP2A in young EPCs at first showed upregulated phospho‐p38 and phospho‐JNK, consistent with the same findings of decreased PP2A and increased phospho‐p38/phospho‐JNK in senescent EPCs after serial passage. In addition, in miRNA‐409‐3p‐overexpressed young EPCs, PP2A protein was downregulated and, in parallel, time series analysis showed p38 and JNK was significantly activated by phosphorylation, especially 48 h after overexpression of miRNA in EPCs, at which time point angiogenic activities were inhibited. Surprisingly, in miRNA‐409‐3p‐overexpressed young EPCs, the inhibited angiogenic activities including proliferation, migration and tube formation activities were significantly improved and reversed by adding the p38 inhibitor but not by the JNK inhibitor. Therefore, p38 pathway was closely involved in this senescence‐related EPC biology. The p38 pathway was reported as the third major signalling cassettes of the mitogen‐activated protein kinase (MAPK) signalling pathway. p38 MAPK, the activity of which is mainly regulated by phosphorylation‐dephosphorylation mechanisms, can respond to various environmental stress stimuli, such as cytokines, and is involved in cell differentiation, cell migration and senescence.^34^, ^35^ Ageing‐related activation of the p38 MAPK pathway in muscle stem cells, impaired muscle regeneration.^36^, ^37^ In addition, p38 MAPK regulated angiogenesis and biological function of endothelial progenitor cells.^38^, ^39^, ^40^ In the literature, there were few studies reporting the regulation of microRNA and p38 MAPK in EPCs. In 2015, a report showed that dysregulated microRNA‐26a was associated with impaired EPC function via the p38 MAPK/VEGF pathway.^41^ In the present study, hsa‐miR‐409‐3p were upregulated in senescent EPCs and led to decreased angiogenesis. The regulation involved PP2A and p38 signalling pathways.

Vascular endothelial growth factor (VEGF), which contains at least 7 members, regulates both angiogenesis and vasculogenesis.^42^ VEGF‐A and its receptors VEGFR‐1 and VEGFR‐2 play major roles in physiological and pathological angiogenesis. In the present study, although the regulation via hsa‐miR‐409‐3p/PP2A/p‐p38 still existed when exogenous VEGF was added to the siPP2A‐treated EPCs, the significant change in protein expression of PP2A and p‐p38 in the presence of exogenous VEGF became insignificant. Moreover, after adding exogenous VEGF, the change of p‐JNK protein expression, which was finally proved not to be a key protein involved in hsa‐miR‐409‐3p signalling, became minimal. We also noted that when the miRNA‐409‐3p‐overexpressed EPCs were treated with exogenous VEGF, a decreased trend of PP2A expression existed but became insignificant. These findings implied that exogenous VEGF affected this hsa‐miR‐409‐3p‐dominated signalling pathway. In 2020, investigators found that the B55α subunit (PPP2R2A)/PP2A complex restrains prolyl hydroxylase 2 activity, promoting endothelial cell (EC) survival, and furthermore dephosphorylates p38, altogether protecting ECs against cell stress occurring. They suggested the use of PP2A inhibitors as potent antiangiogenic drugs targeting specifically nascent blood vessels with a mode of action complementary to VEGFR‐targeted therapies.^43^ In 2021, PP2A mediating Yes‐associated protein activation in ECs upon VEGF stimulation and matrix stiffness was found.^22^ Both of the above findings demonstrated the relationship between VEGF and PP2A, which could explain the regulatory relationship involved in the present study. Taken together, hsa‐miR‐409‐3p/PP2A/p‐p38 signalling was involved in human EPC senescence and partially affected by VEGF in this senescence‐related signalling via PP2A.

Based on the findings of hsa‐miR‐409‐3p in human EPC senescence in vitro, we further tested whether the findings can be extrapolated to human ageing in vivo and reflected in the PBMCs. In the literature, studies involving microRNA regulation of endothelial lineages or EPC senescence/ageing were not in a dearth. Reduced expression of miRNA‐130a was reported to promote endothelial cell senescence and led to age‐dependent impairment of neovascularization.^44^ MiRNA‐126 was found downregulated in senescent endothelial cells and regulated angiogenesis in replicative endothelial senescence.^45^ MiRNA‐34a induced EPC senescence and impaired angiogenetic functions.^44^ Overexpression of miRNA‐10A* and miRNA‐21 caused EPC senescence via suppressing high‐mobility group A2.^46^ These examples suggested that the miRNAs regulated senescence of endothelial lineages/EPCs also influenced angiogenesis. The results of present study of hsa‐miR‐409‐3p were consistent with the findings of above‐mentioned reports. On the other hand, miRNAs were differentially expressed during ageing and targeted hundreds of transcripts to modulate various age‐associated cardiovascular processes and pathologies.^47^, ^48^ Age is a critical risk factor for the development of CVD and a leading cause of death in the older population.^49^ In our report in which PBMCs were collected from participants with relatively balanced characteristics regardless of age, hsa‐miR‐409‐3p expression in PBMCs of the old group was found to be significantly increased, compared to the PBMCs of young group. Additionally, the age‐related expression of hsa‐miR‐409‐3p was independent of gender. Our data transcend previous studies by showing the translational impact on the human body.

There are limitations to this study. First, human blood samples of all experiments used here were from Taiwanese and the number of participants is relatively small. Second, the blood donors were mainly volunteers and staff in the hospital, whose lifestyle may be healthier than the general population. Additionally, the signalling mechanisms derived from human EPCs may not reappear in PBMCs in which the clinical implication of hsa‐miR‐409‐3p as an ageing marker stood for. These limitations should be borne in mind and addressed in future research.

In summary, our results indicate that hsa‐miR‐409‐3p is a novel epigenetic modulator. It was involved in the regulation of senescence‐associated signalling pathways in human EPCs by participating in activating downstream PPP2CA gene, downregulating PP2A protein expression, subsequently upregulating phospho‐p38 and finally causing dysfunctional angiogenesis of EPCs. Moreover, our studies demonstrated that hsa‐miR‐409‐3p expression in human PBMCs was significantly upregulated in the elderly regardless of gender. The results highlight the clinical potential of human PBMC‐derived hsa‐miR‐409‐3p as a biomarker in the detection of human ageing.

## AUTHOR CONTRIBUTIONS

**Yi‐Nan Lee:** Data curation (equal); formal analysis (equal); investigation (equal). **Yih‐Jer Wu:** Investigation (equal); project administration (equal); supervision (equal). **Hsin‐I Lee:** Data curation (equal); formal analysis (equal); methodology (equal); resources (equal); software (equal). **Hsueh‐Hsiao Wang:** Formal analysis (equal); investigation (equal). **Chung‐Lieh Hung:** Investigation (equal); visualization (equal). **Chiung‐Yin Chang:** Formal analysis (equal); methodology (equal). **Yen‐Hung Chou:** Project administration (equal); validation (equal); visualization (equal). **Ting‐Yi Tien:** Methodology (equal); project administration (equal); validation (equal). **Chun‐Wei Lee:** Validation (equal); visualization (equal). **Chao‐Feng Lin:** Validation (equal); visualization (equal). **Cheng‐Huang Su:** Conceptualization (lead); funding acquisition (lead); supervision (lead); writing – original draft (lead); writing – review and editing (lead). **Hung‐I Yeh:** Supervision (equal); validation (equal); writing – review and editing (equal).

## CONFLICT OF INTEREST STATEMENT

The authors declare no conflict of interest associated with this manuscript.

## Supporting information


Figure S1.

Figure S2.

Figure S3.

Figure S4.

## Data Availability

The data that support the findings of this study will be available from the corresponding author upon reasonable request.
