# Supplementary figures and images for: Hsa‐miR‐409‐3p regulates endothelial progenitor senescence via PP2A‐P38 and is a potential ageing marker in humans
Source: J Cell Mol Med. 2023 Feb 9;27(5):687–700. doi: 10.1111/jcmm.17691 (PMC9983318; doi:10.1111/jcmm.17691)

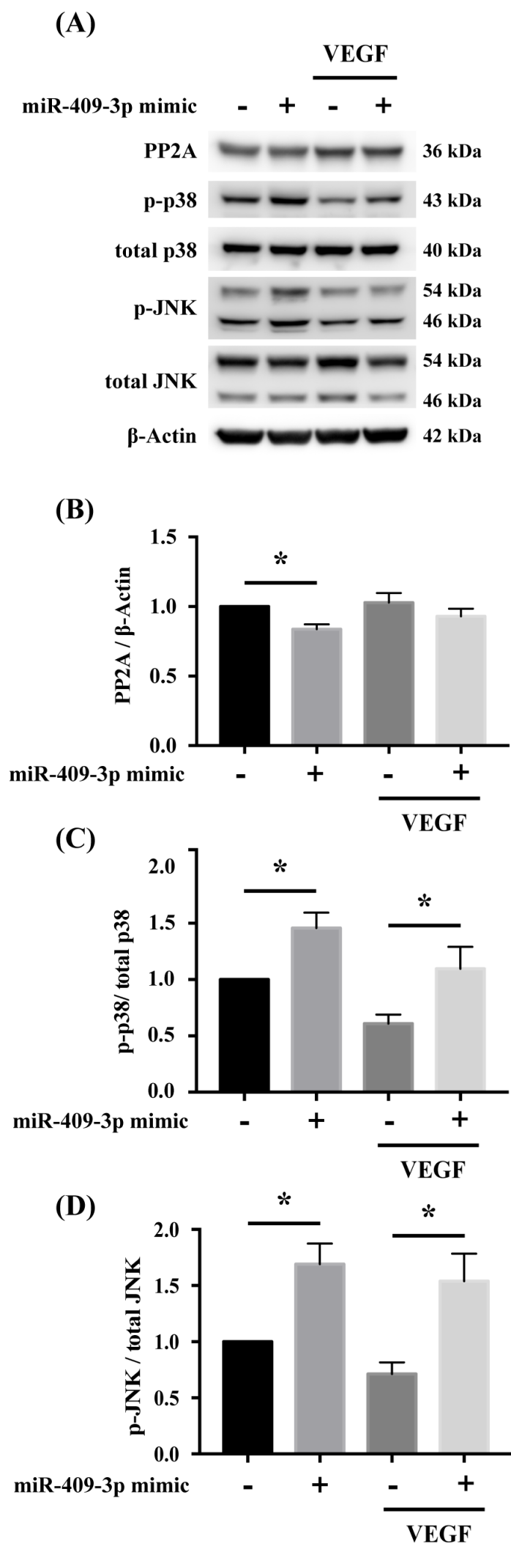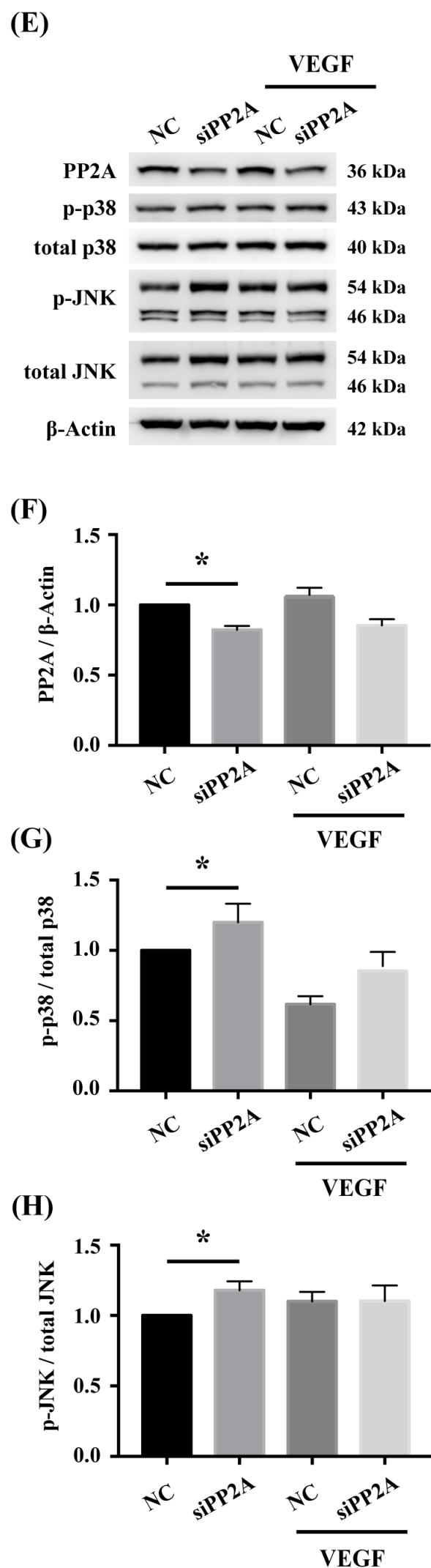

Supplement: Supplementary file 1 — Figure S1. [file JCMM-27-687-s004.pdf]

(A)

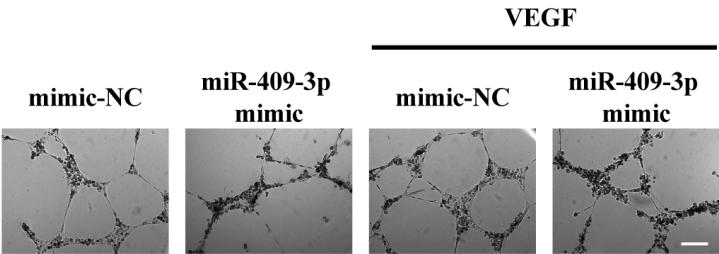

(B)

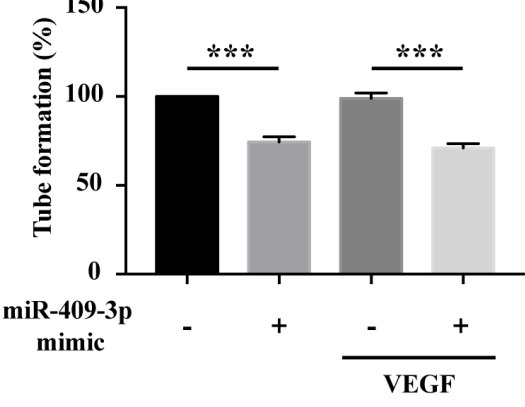

(C)

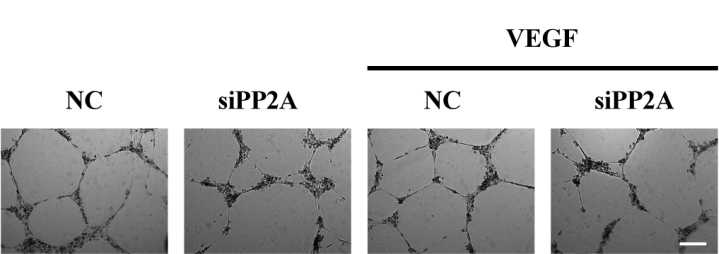

(D)

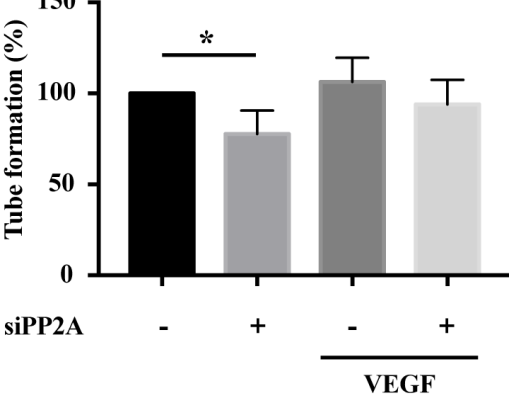

Supplement: Supplementary file 2 — Figure S2. [file JCMM-27-687-s002.pdf]

**(A)**

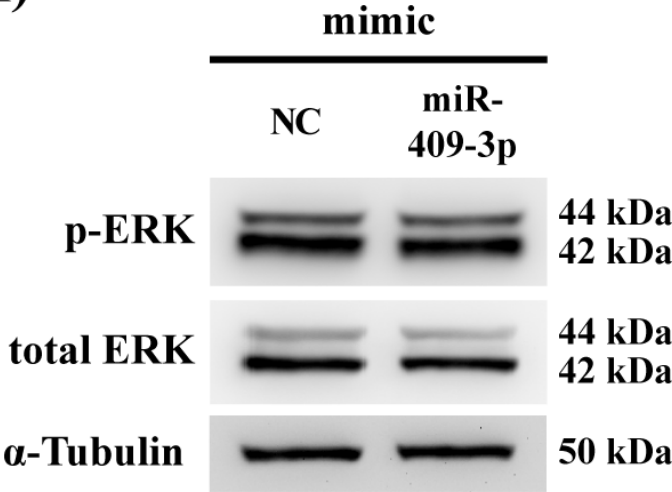

**(B)**

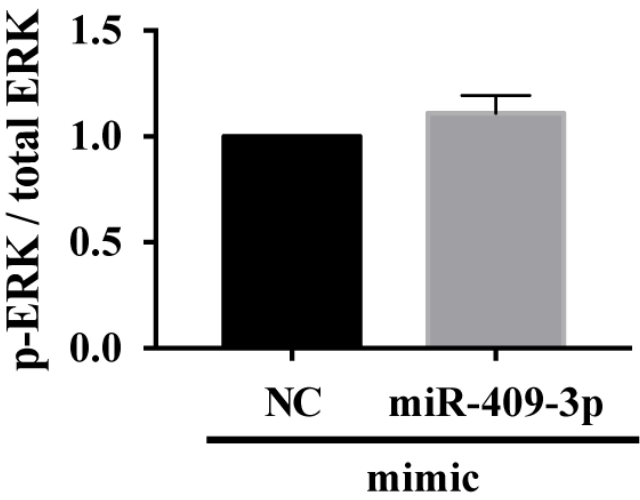

Supplement: Supplementary file 3 — Figure S3. [file JCMM-27-687-s001.pdf]

(A)

mimic-  
NC

miR-409-3p  
mimic

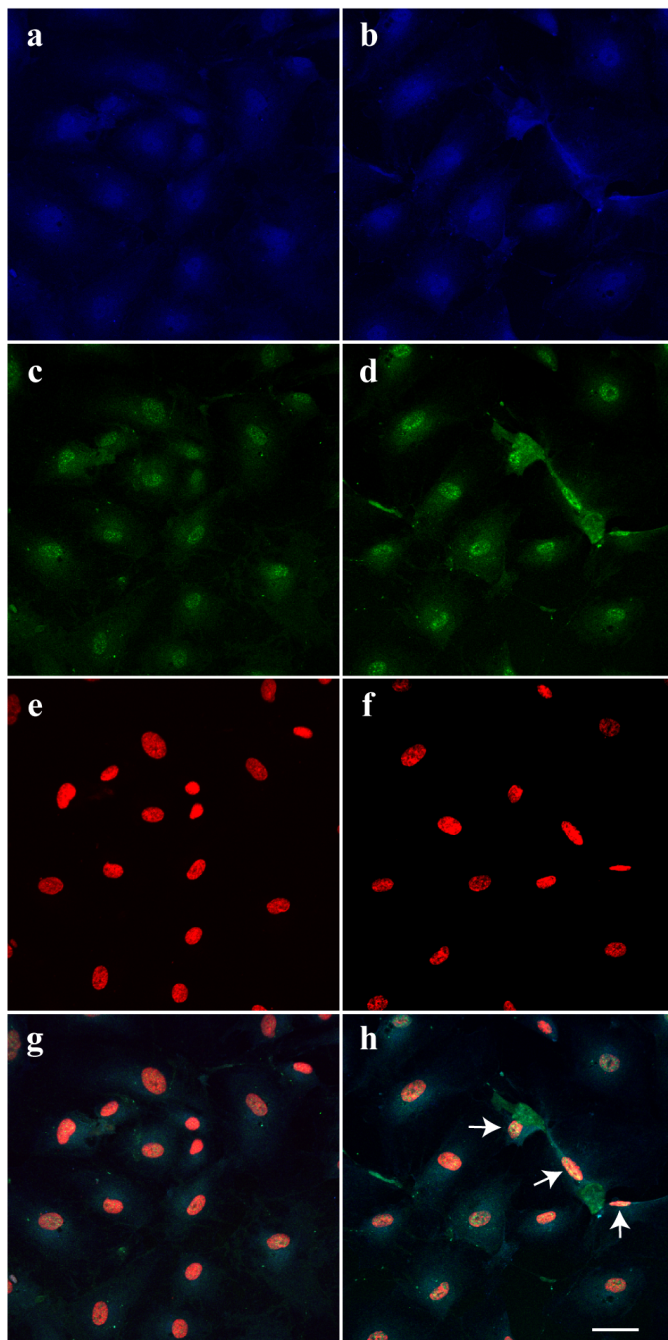

(B)

mimic-  
NC

miR-409-3p  
mimic

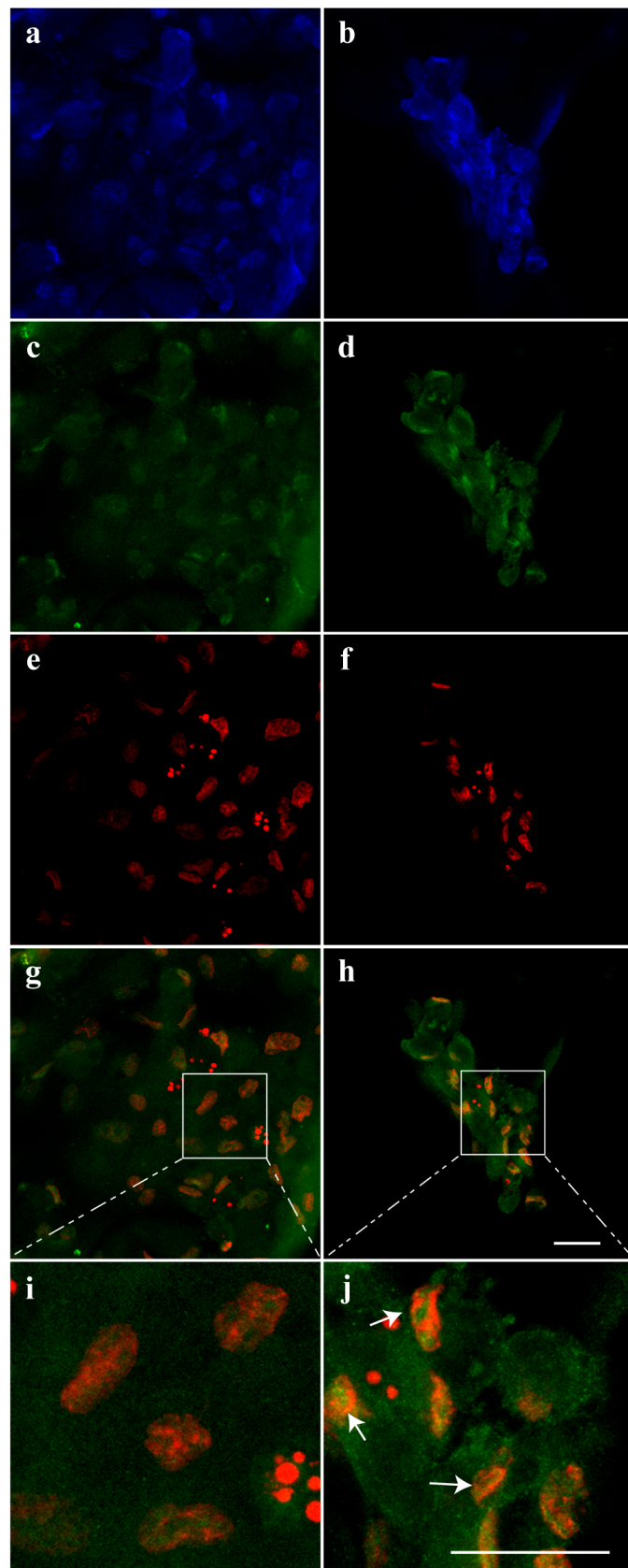

Supplement: Supplementary file 4 — Figure S4. [file JCMM-27-687-s003.pdf]
